# Conservative non-surgical management of penetrating cardiac injury with three retained metal nails: a case report

**DOI:** 10.1093/ehjcr/ytaf380

**Published:** 2025-08-31

**Authors:** Ramtin Khanipour, Saro Mazmanian, Andrew Jung, Ali Dahhan

**Affiliations:** Division of Cardiology, HCA Healthcare—HCA Florida Bayonet Point/USF Morsani College of Medicine, 14000 Fivay Rd, Hudson, FL 34667, USA; Division of Cardiology, HCA Healthcare—HCA Florida Bayonet Point/USF Morsani College of Medicine, 14000 Fivay Rd, Hudson, FL 34667, USA; Division of Cardiology, HCA Healthcare—HCA Florida Bayonet Point/USF Morsani College of Medicine, 14000 Fivay Rd, Hudson, FL 34667, USA; Division of Cardiology, HCA Healthcare—HCA Florida Bayonet Point/USF Morsani College of Medicine, 14000 Fivay Rd, Hudson, FL 34667, USA

**Keywords:** Penetrating cardiac injury, Retained foreign body, Infective endocarditis, Surgical approach, Conservative approach

## Abstract

**Background:**

Traumatic penetrating cardiac injuries are usually immediately fatal. The management approach (surgical vs. conservative) in surviving patients can be challenging. A conservative approach is appropriate in selective cases; however, long-term complications should be considered.

**Case summary:**

A frail 83-year-old male who had undergone coronary artery bypass grafting surgery 20 years ago presented with encephalopathy. He had persistent bacteraemia. Chest computed tomography revealed three metal nails penetrating the anterior thoracic wall through the myocardium. Later, we learned that he had attempted suicide 2 years ago by shooting himself in the chest using a nail gun. Conservative non-surgical approach was pursued at that time given his multiple comorbidities. We adopted a conservative approach again. He survived for additional 6 months on chronic suppressive antimicrobial therapy but ultimately passed away from pneumonia.

**Discussion:**

The favourable outcome in this patient underscores the role of a conservative approach in selective cases. Retained foreign bodies pose a risk of infective endocarditis; therefore, empirical chronic antimicrobial therapy and tetanus vaccination should be considered.

Learning points

- A conservative, non-surgical approach can be considered in penetrating cardiac injuries if the patient is haemodynamically stable, after careful evaluation of the risks and benefits.
- Chronic suppressive antimicrobial therapy and tetanus vaccination should be considered in the management of retained foreign bodies.

## Introduction

Traumatic penetrating cardiac injuries are typically immediately fatal. Most patients are either found dead at the scene or present with severe haemodynamic instability. Even among those few who survive and reach the hospital, the mortality rate remains high.^1^ Therefore, immediate surgical intervention is the cornerstone in management. However, a rare subset of patients can be haemodynamically stable and therefore pose a significant management challenge, particularly in determining whether to pursue a surgical or conservative approach. A conservative approach can be pursued in selective cases, but clinicians should carefully weigh the potential long-term complications of retained foreign bodies.

## Summary figure

**Figure:**
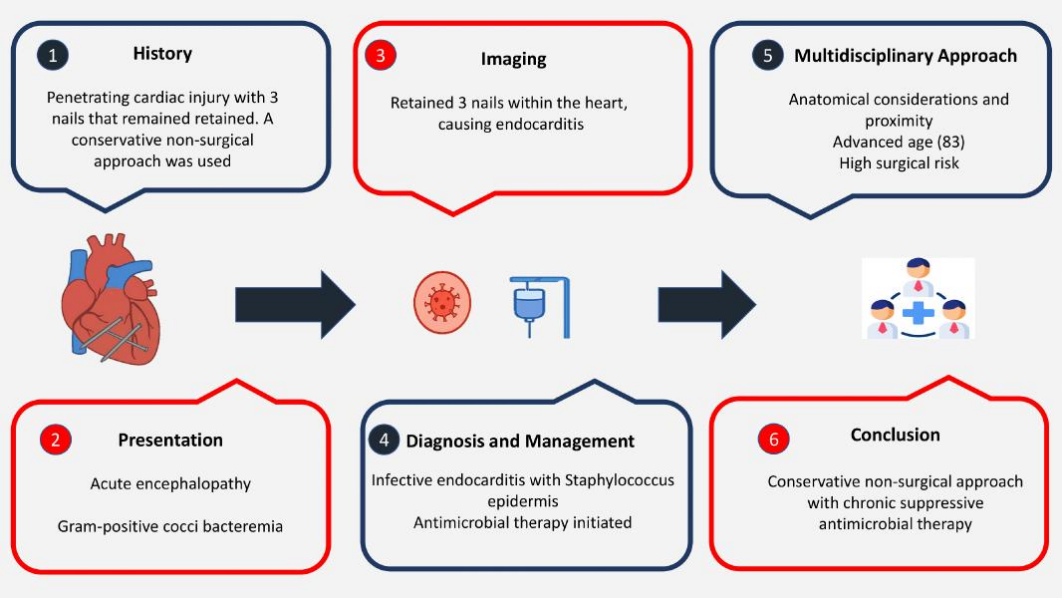


## Case presentation

A frail 83-year-old man presented to the emergency department with acute encephalopathy. His past medical history included remote coronary artery bypass grafting (CABG) surgery performed 20 years ago, mild-to-moderate aortic stenosis, hypertension, dyslipidaemia, diabetes mellitus, and depression. His temperature was 37.4°C, heart rate was 71 b.p.m., blood pressure was 149/61 mmHg, and respiratory rate was 17 breaths/min. He was awake but confused, with no focal neurological deficits.

**Figure 1:**
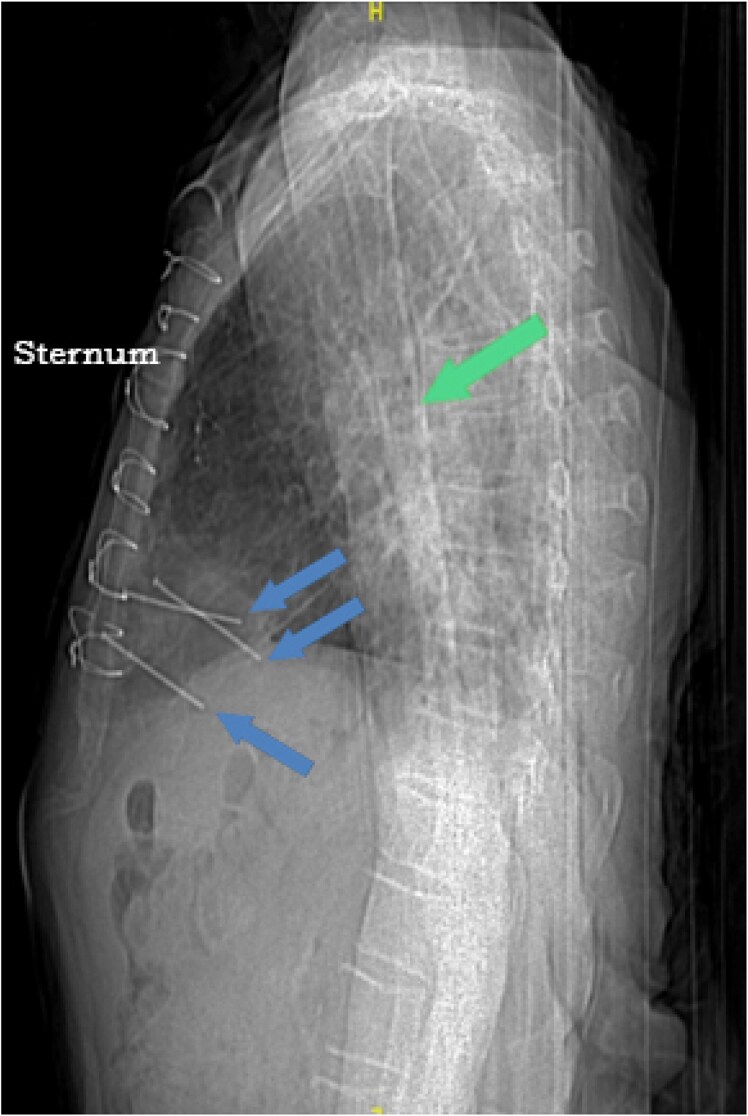
Lateral chest radiogram illustrating three nails (blue arrows) penetrating the thoracic cavity into the anterior mediastinum. The green arrow marks the humerus (the patient could not place his arms above his head).

Initial laboratory work-up did not reveal any significant abnormalities except for a white blood count of 11 000/μL. Electrocardiography showed sinus rhythm with non-specific T wave changes. Chest X-ray showed three linear metallic objects penetrating the anterior thoracic wall into the middle mediastinum (*Figure 1*). Transthoracic echocardiography showed a single linear echodensity attached to the interventricular septum (IVS), extending through the left ventricular (LV) cavity. The tip of the echodensity seemed to interact with the tip of the posteromedial papillary muscle which itself had an echodensity attached to it (Supplementary material online, *Video S1*). Blood cultures later grew *Staphylococcus epidermidis*.

Transoesophageal echocardiography demonstrated a single linear echodensity attached to the IVS and confirmed its interaction with the tip of the posteromedial papillary muscle. There was also a mobile echodensity measuring 1.0 cm × 1.5 cm attached to the tip of the posteromedial papillary muscle consistent with a vegetation (Supplementary material online, *Video S2*). Mitral regurgitation was only mild. Intravenous antimicrobial therapy was initiated.

After further investigation, those foreign bodies later turned out to be retained metal nails. We discovered that 2 years ago, the patient had attempted suicide by intentionally shooting himself in the chest using a nail gun. Three nails had penetrated his thorax and penetrated the myocardium. At that time, given the absence of symptoms, haemodynamic stability, multiple comorbidities, and prior sternotomy with CABG using the left internal mammary artery, a conservative non-surgical management approach was pursued, and he was discharged home without antimicrobial therapy. There was no documentation that tetanus vaccination was administered or considered.

**Figure 2:**
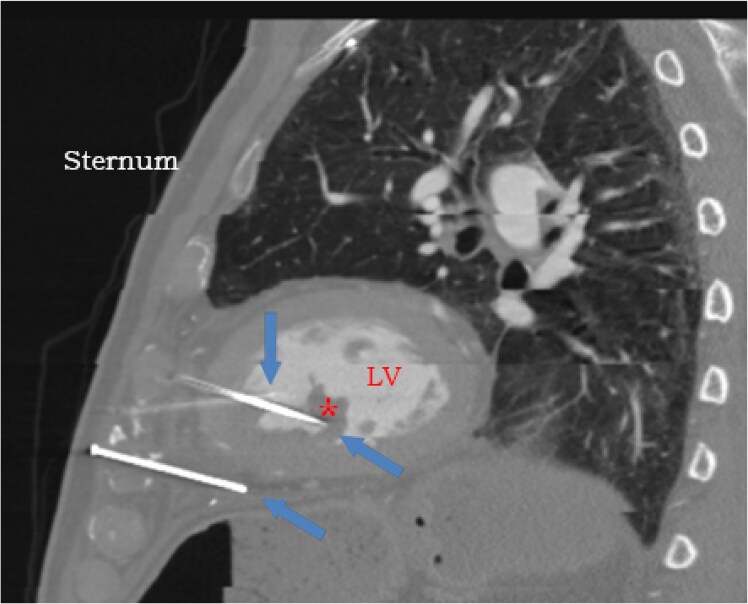
Computed tomographic angiography of the heart showing three nails penetrating thoracic cavity, two of which penetrate through the heart. The left ventricular (LV) cavity is noted. The tip of the first nail (top blue arrow) terminates in the sub-endocardium of the mid interventricular septum. The tip of second nail (middle blue arrow) appears to terminate in the posteromedial papillary muscle (red asterisk). The third nail (bottom blue arrow) terminates adjacent to the inferior wall of the left ventricular myocardium.

**Figure 3:**
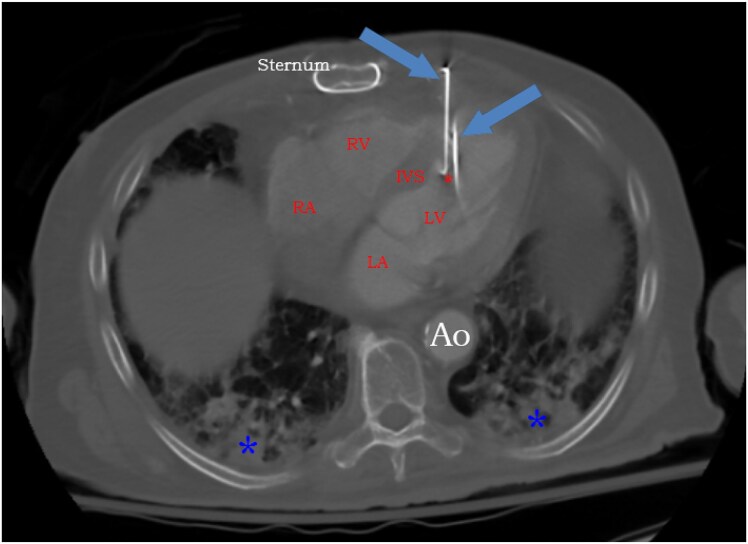
Computed tomographic angiography of the chest showing two nails penetrating through the heart. The tip of one of the nails (left blue arrow) terminates in the sub-endocardium of the mid interventricular septum (IVS). The tip of the other nail (right blue arrow) appears to terminate in the posteromedial papillary muscle (red asterisk). The third nail is not seen in this plane. Infiltrates are noted in the lower lobes of the lungs bilaterally (blue asterisks). Ao, descending thoracic aorta; LA, left atrium; LV, left ventricle; RA, right atrium; RV, right ventricle.

**Figure 4:**
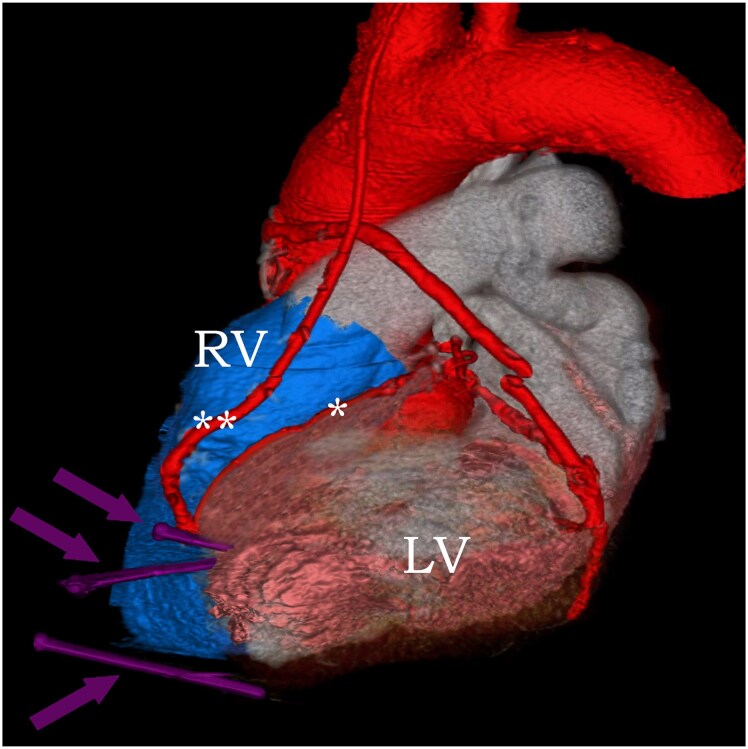
3D computed tomographic construction of the heart. Three nails are seen (purple arrows), two of them penetrate through the heart and the third one is adjacent to the inferior wall. The left anterior descending (LAD) artery (*) travels between the left ventricle (LV) and right ventricle (RV) and the left internal mammary arterial graft (**) is anastomosed to it. The two nails penetrate the heart close to both the left anterior descending artery and left internal mammary artery.

Cardiac computed tomographic angiography confirmed the presence of three metal nails penetrating the anterior thoracic cavity into the myocardium. The tips of two of those nails terminated within the LV cavity and one of them terminated within the posteromedial papillary muscle. The third nail terminated adjacent to the inferior wall of the LV (*Figures 2* and *3*). Two of those three nails were very close to the left anterior descending (LAD) artery and left internal mammary artery that was anastomosed to it (*Figure 4*). The location of the nails was unchanged compared to 2 years ago. After multidisciplinary team discussions, we determined that pursuing a conservative approach was again appropriate. Bacteraemia resolved after 6 weeks of intravenous antimicrobial therapy. Tetanus vaccination was not needed at that point. He was discharged home on chronic suppressive antimicrobial therapy.

## Follow-up

The patient did well, but passed away 6 months later due to pneumonia and its complications.

## Discussion

The majority of patients who sustain penetrating cardiac injuries are haemodynamically unstable and immediate surgical intervention should intuitively be pursued without delays.^1,2^ However, some patients remain haemodynamically stable, and these pose a significant management challenge.^3–7^ In such selective cases, a thorough assessment of the risks and benefits of both surgical and conservative approaches should be performed. The decision should be based on symptoms, clinical status, haemodynamic consequences, comorbidities, and both short- and long-term prognosis.^3^

Our patient sustained a self-inflicted penetrating cardiac injury involving three metal nails (*Summary Figure*). Some of the nails were even close to the LAD artery and its bypass graft (*Figure 4*); yet, he did survive such an injury. He did fairly well with a conservative approach for 2 years, even without suppressive antimicrobial therapy. However, the retained nails within the myocardium eventually led to infective endocarditis and bacteraemia. Given his frailty and multiple comorbidities, a conservative approach was pursued again, and he responded favourably to chronic suppressive antimicrobial therapy. Nonetheless, he ultimately passed away due to pneumonia and its complications. It is important to note that pneumonia in elderly patients with multiple comorbidities carries a higher risk of morbidity and mortality. This further supports that the decision to pursue conservative approach in this frail patient was appropriate.

With the paucity of the available literature, determining the optimal conservative approach remains challenging. Clinicians should carefully consider the risks associated with retained cardiac foreign bodies, including bacteraemia, infective endocarditis, embolization, and arrhythmias. It might have been reasonable to consider chronic suppressive antimicrobial therapy after the initial injury 2 years ago. Tetanus vaccination should also have been administered. Antiarrhythmic therapy may be indicated if clinically significant arrhythmias are present. Certain foreign bodies, such as bullets, may pose the risk of embolization. Retrieval of such foreign bodies using percutaneous approach can be feasible in selective cases after considering the location of the foreign body and the risk of embolization.^8–10^

## Conclusion

The management of haemodynamically stable patients with penetrating cardiac injuries with retained foreign bodies remains challenging. It should be determined based on the clinical status, risks/benefit ratio, and overall prognosis. A conservative approach may be appropriate in selective cases and should involve chronic suppressive antimicrobial therapy and tetanus vaccination.

## Lead author biography


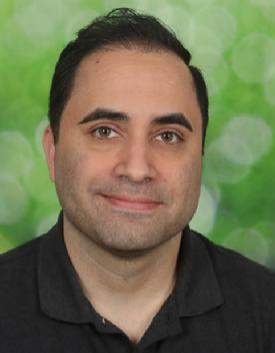


Dr Ramtin Khanipour obtained his Bachelor of Science degree in cell and molecular from California State University of Fullerton. He completed his medical education at Lincoln Memorial University in the state of Tennessee. He recently finished his internal medicine residency at HCA Florida Bayonet Point Hospital-University of South Florida Medical Center. He will be applying to cardiology fellowship in the United States of America. His areas of interest include preventive cardiology and structural heart disease.

## Supplementary Material

ytaf380_Supplementary_Data

## Data Availability

The data underlying this article will be shared on reasonable request to the corresponding author.

## References

[1] O'Connor J, Ditillo M, Scalea T. Penetrating cardiac injury. J R Army Med Corps 2009;155:185–190. 20397356 10.1136/jramc-155-03-02

[2] Schreyer C, Schulz-Drost S, Markewitz A, Breuing J, Prediger B, Becker L, et al Surgical management of chest injuries in patients with multiple and/or severe trauma- a systematic review and clinical practice guideline update. Eur J Trauma Emerg Surg 2024;50:2061–2071. 38888790 10.1007/s00068-024-02556-1 PMC11599403

[3] Actis Dato GM, Arslanian A, Di Marzio P, Filosso PL, Ruffini E. Posttraumatic and iatrogenic foreign bodies in the heart: report of fourteen cases and review of the literature. J Thorac Cardiovasc Surg 2003;126:408–414. 12928637 10.1016/s0022-5223(03)00399-4

[4] Asensio JA, Soto SN, Forno W, Roldan G, Petrone P, Salim A, et al Penetrating cardiac injuries: a complex challenge. Injury 2001;32:533–543. 11524085 10.1016/s0020-1383(01)00068-7

[5] Ball CG, Lee A, Kaminsky M, Hameed SM. Technical considerations in the management of penetrating cardiac injury. Can J Surg 2022;65:E580–E592. 36302130 10.1503/cjs.008521 PMC9451503

[6] Hromalik LJ, Wall MJ, Mattox KL, Tsai PI. Penetrating cardiac injury: a narrative review. Mediastinum 2023;7:15. 37261091 10.21037/med-22-18 PMC10226890

[7] Lee C, Jebbia M, Morchi R, Grigorian A, Nahmias J. Cardiac trauma: a review of penetrating and blunt cardiac injuries. Am Surg 2025;91:423–433. 39661455 10.1177/00031348241307400

[8] Gaylord GM, Johnsrude IS. Split 24-F Amplatz dilator for percutaneous extraction of an intravascular bullet: case report and technical note. Radiology 1989;170:888–889. 2644664 10.1148/radiology.170.3.2644664

[9] Chew JD, Nicholson GT, Mettler BA, Doyle TP. Percutaneous removal of intravascular pellet following penetrating cardiac trauma. Pediatr Cardiol 2018;39:191–194. 28780711 10.1007/s00246-017-1689-3

[10] McLaughlin RL, Analitis S, VanVleet S, Pederson R. Right ventricular gunshot wound with retrograde embolization. J Trauma Nurs 2008;15:123–125. 18820560 10.1097/01.JTN.0000337154.16088.6d

